# Prospective Clinical Study of Non-Invasive Intracranial Pressure Measurements in Open-Angle Glaucoma Patients and Healthy Subjects

**DOI:** 10.3390/medicina56120664

**Published:** 2020-11-30

**Authors:** Mantas Deimantavicius, Yasin Hamarat, Paulius Lucinskas, Rolandas Zakelis, Laimonas Bartusis, Lina Siaudvytyte, Ingrida Janulevicienė, Arminas Ragauskas

**Affiliations:** 1 Health Telematics Science Institute, Kaunas University of Technology, 51423 Kaunas, Lithuania; mantas.deimantavicius@ktu.lt (M.D.); paulius.lucinskas@ktu.lt (P.L.); rolandas.zakelis@ktu.lt (R.Z.); laimonas.bartusis@ktu.lt (L.B.); telematics@ktu.lt (A.R.); 2 Eye Clinic, Lithuanian University of Health Sciences, 50161 Kaunas, Lithuania; lynciuke@gmail.com (L.S.); ingrida.januleviciene@kaunoklinikos.lt (I.J.)

**Keywords:** primary open angle glaucoma, normal-tension glaucoma, high-tension glaucoma, intracranial pressure, non-invasive ICP measurement

## Abstract

*Background and Objective:* Glaucoma is a progressive optic neuropathy in which the optic nerve is damaged. The optic nerve is exposed not only to intraocular pressure (IOP) in the eye, but also to intracranial pressure (ICP), as it is surrounded by cerebrospinal fluid in the subarachnoid space. Here, we analyse ICP differences between patients with glaucoma and healthy subjects (HSs). *Materials and Methods:* Ninety-five patients with normal-tension glaucoma (NTG), 60 patients with high-tension glaucoma (HTG), and 62 HSs were included in the prospective clinical study, and ICP was measured non-invasively by two-depth transcranial Doppler (TCD). *Results:* The mean ICP of NTG patients (9.42 ± 2.83 mmHg) was significantly lower than that of HSs (10.73 ± 2.16 mmHg) (*p* = 0.007). The mean ICP of HTG patients (8.11 ± 2.68 mmHg) was significantly lower than that of NTG patients (9.42 ± 2.83 mmHg) (*p* = 0.008) and significantly lower than that of HSs (10.73 ± 2.16 mmHg) (*p* < 0.001). *Conclusions:* An abnormal ICP value could be one of the many influential factors in the optic nerve degeneration of NTG patients and should be considered as such instead of just being regarded as a “low ICP”.

## 1. Introduction

Glaucoma is a progressive optic neuropathy leading to irreversible vision loss, and is also characterised by structural degeneration of the optic nerve head. The lamina cribrosa (LC), located deep within the optic nerve head [1], is a sieve-like structure in the posterior portion of the sclera that allows optic nerve fibres to exit from the eye [2,3]. The LC plays an important role as a barrier between intraocular pressure (IOP) and intracranial pressure (ICP) [4,5]. Elevated IOP was formerly considered to be the main risk factor in the development of glaucoma, however, elevated IOP is not always present in all forms of glaucoma [5,6]. Primary open-angle glaucoma, which is the most common type of glaucoma worldwide, can be clinically classified into two subgroups: high-tension glaucoma (HTG), in which elevated IOP plays a major role, and normal-tension glaucoma (NTG), in which IOP is within the normal range [7]. Three studies where direct measurements of ICP were performed have demonstrated that ICP is significantly lower in NTG patients than in HTG patients or healthy subjects (HSs) [6,8,9,10], suggesting that ICP has an impact on glaucoma [11,12]. However, two studies have contradicted this idea and reported no significant differences in ICP between NTG patients and healthy controls [8,13], suggesting that the ICP regulatory system is not the major component of the NTG pathophysiology [13].

ICP can be monitored in a limited group of patients due to the invasive nature of the measurement. However, a non-invasive measurement method of ICP which has clinically acceptable accuracy, precision, and diagnostic reliability can overcome this limitation [14,15,16] and can be applied to a wider range of patient groups. The method is based on the principles of a non-invasive arterial blood pressure measurement and uses two depth transcranial Doppler (TCD) ultrasonography to assess blood flow velocity of the ophthalmic artery (OA) during a gradual externally applied pressure (Pe) over a closed eyelid that is transmitted to the eye and orbital (peri-ocular) tissues [17,18].

The aim of this prospective clinical study was to assess ICP differences between glaucoma patients (HTG and NTG) and healthy subjects.

## 2. Materials and Methods

The prospective clinical study was performed at the eye clinic of the Lithuanian University of Health Sciences. The study was approved by the Kaunas Regional Biomedical Research Ethics Committee (No. BE-2-41, date: 2013-09-03), and all participants provided written informed consent, according to the Declaration of Helsinki.

Glaucoma patients (HTG and NTG) and healthy subjects were enrolled in the study. Only one eye per subject was used for ICP measurement. The eye with greater glaucomatous damage was selected in the glaucoma patient, while the eye was selected randomly in healthy subjects. The inclusion criteria were as follows: clinical diagnosis of glaucoma confirmed by an ophthalmologist, characteristic optic nerve head changes present, and visual field loss consistent with glaucoma. A neurologist examined all patients to exclude neurological disorders that could affect ICP. The exclusion criteria were as follows: pregnant or nursing women, patients with uncontrolled systemic diseases and patients with a history of allergy to local anaesthetics, and orbital/ocular trauma or other diseases that could bias study results. Current medical treatment was continued with the exception of oral carbonic anhydrase inhibitors due to their known effects on ICP. In the case of HSs, volunteers with no history of glaucoma or other diseases that could bias the results were included. Details of inclusion, exclusion criteria, and the study population are shown in Table 1.

The non-invasive measurement method of the absolute value of ICP, which does not need an individual patient-specific calibration, is based on the two-depth high-resolution TCD technique for simultaneously measuring blood flow velocity in the intracranial and extracranial segments of the ophthalmic artery (OA) [14]. A 2-MHz ultrasonic transducer is installed into the head frame together with an air-filled toroidal-shaped soft plastic pressure cuff. Due to the nature of non-compressible orbital tissues as well as the segmentation by the dura mater, the externally applied pressure (Pe) via pressure cuff is transmitted to extracranial OA, but not the intracranial OA. The intracranial segment of the OA is compressed by ICP, and the extracranial segment of the OA is compressed by the externally applied pressure. Blood flow parameters, such as flow velocity pulsations in both OA segments, are approximately equal when Pe = ICP. In this study, Pe was gradually increased from 0 to 20 mmHg by 4 mmHg steps. All subjects were in a supine position during the procedure. The duration of the measurement procedure was up to 10 min [17]. IOP was measured with a Goldmann applanation tonometer just before the non-invasive ICP measurement procedure. All examinations were performed at daytime between 8 am and 2 pm.

Statistical analysis was performed using IBM SPSS Statistics software (version 23.0; IBM Corporation, Armonk, NY, USA). All variables were defined by methods of descriptive statistics. The analysis of the quantitative variables included the calculation of the mean value (Mean) and standard deviation (SD). The Kolmogorov–Smirnov test for the testing of data normality distribution was used for the analysis of all three groups: HTG, NTG, and HSs. The one-way ANOVA test and Tukey multiple comparisons test were performed between subject groups.

## 3. Results

**Figure 1:**
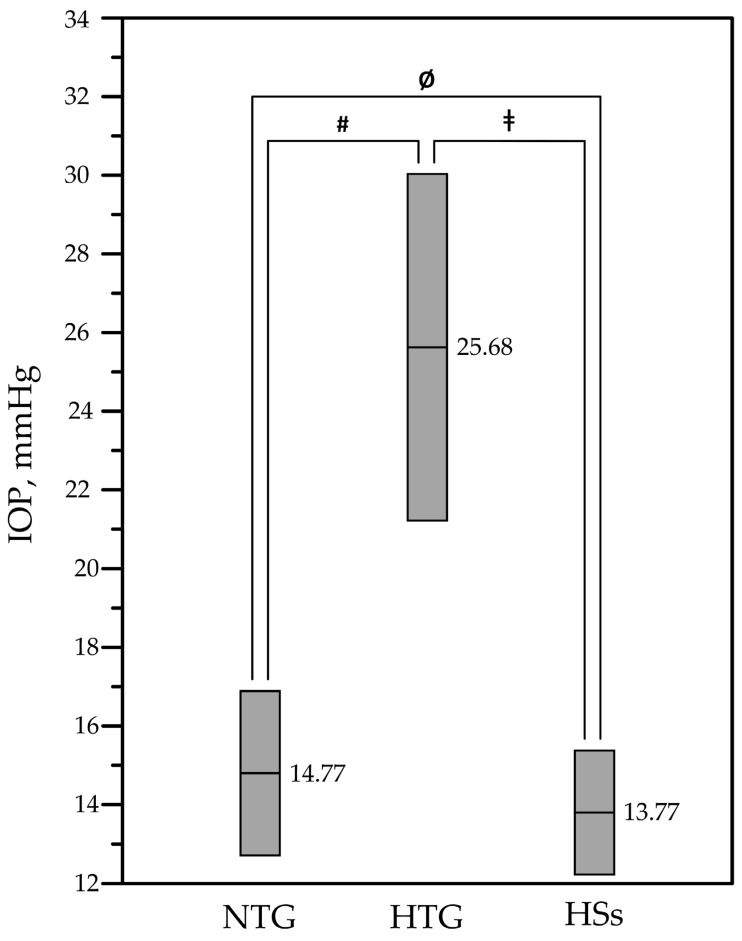
Mean IOP values with standard deviation for NTG, HTG, and HSs. NTG: normal-tension glaucoma patients; HTG: high-tension glaucoma patients; HSs: healthy subjects. Ø—the difference is statistically significant (*p* < 0.05) comparing the means of the IOP data of NTG patients and HSs. #—the difference is statistically significant (*p* < 0.05) comparing the means of the IOP data of NTG patients and HTG patients. ǂ—the difference is statistically significant (*p* < 0.05) comparing the means of the IOP data of HTG patients and HSs.

Two-hundred-seventeen subjects, of which 95 were patients with NTG, 60 were patients with HTG, and 62 were HSs, were included in the statistical analysis of this study, after the exclusion criteria were applied. Demographic data and medication of the subjects are depicted in Table 2. NTG patients had significantly (*p* < 0.05) lower IOP compared to HTG patients (Figure 1). HSs had significantly (*p* < 0.05) lower IOP compared to patients with glaucoma (Figure 1).

The Kolmogorov–Smirnov test for the testing of data normality distribution was used for all three groups: NTG, HTG, and HSs. The mean ICP values and tests of data normality are presented in Table 3.

Data did not deviate significantly from the normal distribution, so for the comparison of three independent samples, one-way ANOVA-test was used. Levene’s test for the homogeneity of variances was used. The assumption of equal variance was not rejected (Levene statistic value = 2.343, *p* = 0.1). The average ICP values were found to be different across the groups (*F*(2.214) = 15.315, *p* < 0.001).

**Figure 2:**
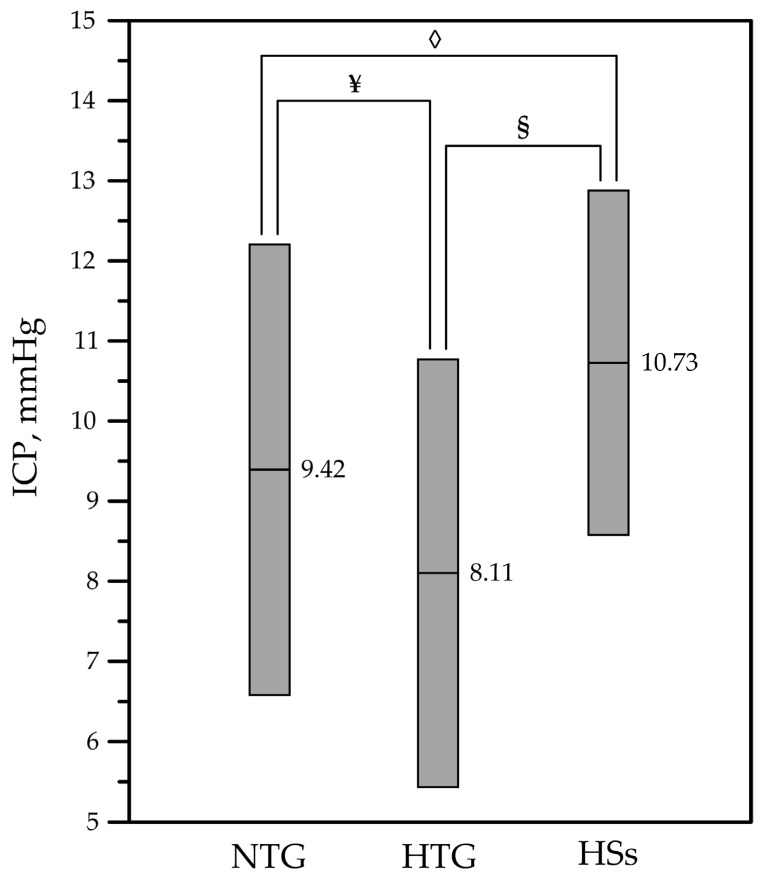
Mean ICP values with standard deviation for NTG, HTG, and HSs. NTG: normal-tension glaucoma patients; HTG: high-tension glaucoma patients; HSs: healthy subjects. ◊—the difference is statistically significant (*p* = 0.007) comparing the means of the ICP data of NTG patients and HSs. ¥—the difference is statistically significant (*p* = 0.008) comparing the means of the ICP data of NTG patients and HTG patients. §—the difference is statistically significant (*p* < 0.001) comparing the means of the ICP data of HTG patients and HSs.

Tukey multiple comparisons performed at the 0.05 significance level found that the mean ICP of NTG patients (9.42 ± 2.83 mmHg) was significantly lower than that of HSs (10.73 ± 2.16 mmHg) (*p* = 0.007). The mean ICP of HTG patients (8.11 ± 2.68 mmHg) was significantly lower than that of NTG patients (9.42 ± 2.83 mmHg) (*p* = 0.008) and significantly lower than that of HSs (10.73 ± 2.16 mmHg) (*p* < 0.001). Results are presented in Figure 2.

## 4. Discussion

Although the potential role of low ICP in the pathogenesis of glaucomatous optic neuropathy was described in the late 1970s [19], the underlying mechanism has remained elusive. Some animal studies have been performed to clarify the association between the cerebrospinal fluid (CSF) pressure and glaucoma [20,21]. A limited number of clinical studies have identified lower CSF pressure in patients with NTG compared to individuals without glaucoma [9,22]. A large number of clinical trials are not available yet due to the invasive nature of CSF pressure measurements. The standard clinical procedure to measure ICP is either to use a lumbar puncture technique or to insert a transducer inside the skull. Thus, in some studies of glaucoma patients, ICP was measured not behind the sieve plate but in the spine by means of lumbar puncture [8,9,22,23]. In this study, we have measured ICP non-invasively closer to lamina cribrosa, and this method allows us to distinguish the pressure in both the intracranial and optic nerve subarachnoid spaces.

In this prospective clinical study that included healthy subjects and patients with glaucoma (NTG and HTG), the mean ICP in HTG patients was significantly lower than in NTG patients and in HSs, while the mean ICP in NTG patients was significantly lower than in HSs. Using the non-invasive method to measure ICP, we calculated the mean ICP value in the NTG patients as being similar to values reported in previous studies [9,22]. In a previous pilot study, the same non-invasive device showed similar mean ICP values for HTG patients and for HSs [24] as those reported in this study. In contrast, the mean ICP of the current study for NTG patients was not similar to the previous pilot study [24]. Furthermore, in our study, we found a large variability in ICP, ranging from 3.21 to 15.79 mmHg in the patients with NTG, 3.37 to 15.17 mmHg in the patients with HTG, and 7.2 to 15.17 mmHg in the case of HSs. The ICP begins to decline progressively after the age of 50 years, with a mean ICP of 10.7 ± 2.6 mmHg at age 60–64 years [25], which is similar to our finding, a mean ICP of 10.73 ± 2.16 mmHg in the case of HSs with a mean age 57.39 ± 10.62 years.

The underlying reason for NTG remains somewhat unclear. A significant percentage of NTG patients have a family history of glaucoma [26], yet NTG is considered a multifactorial disease, and vascular dysregulation could be the key factor in the disease pathway [26]. There is an ongoing debate about disturbed CSF dynamics in the NTG pathway. Some studies have reported increased optic nerve sheath diameters in patients with NTG [23,27], which contradicts the idea of decreased ICP in NTG patients. The conflict might be explained by higher tissue elasticity in such patients and compartmentation of the subarachnoid space by means of disturbed CSF flow [23].

Our prospective clinical study is contradictory to a result obtained in an Asian and in an American population, where the lumbar CSF pressure measurements were significantly lower in NTG patients than in HTG patients [6,9]. Our findings also contradicted two other studies (in Switzerland and in Sweden) where no significant differences in ICP were observed between NTG patients and healthy controls [8,13]. Several reasons could explain the differences between the results obtained in our study and other studies. First, ICP is influenced by body position. The lumbar puncture procedure is generally performed at lateral decubitus position, while non-invasive ICP measurement is taken at the supine position. Second, CSF pressure was measured in-between lumbar vertebrae L3/L4 or L4/L5 in the studies mentioned above, while in our study, it was measured close to the region of interest, the optic nerve. This can influence the measurement result, as a cerebrospinal fluid pathway might not fully communicate because the central nervous system has multiple and rigid subdivisions [26]. The optic nerve compartment syndrome could limit the free flow of CSF [5]. Third, ICP and IOP fluctuate over time, and this makes it difficult to evaluate pressure at a certain time [8,26]. Although the time of measurement is important, we did not compare differences between ICP and IOP in this study. Due to a high number of patients and shortage of staff in the clinic, only one IOP and non-invasive ICP measurement were taken per subject (which took place between 8 am and 2 pm). Next, we used a non-invasive ICP measurement method instead of the invasive lumbar puncture technique, which might represent sample errors yet to be revealed. Also, this study did not include a wash-out period; consequently, hypotensive agents might have affected the ICP value. Also, ICP measurements could be influenced by blood pressure, body mass index, age, and undetermined neurological and systemic disease in different study groups and ethnicities.

## 5. Conclusions

Here, we found that NTG patients had significantly lower ICP compared to HSs, while HTG patients had significantly lower ICP than NTG patients and HSs. The abnormal ICP value on lamina cribrosa could be one of the many factors influencing optic nerve degeneration of NTG patients and should be considered as such instead of being regarded separately as just a “low ICP”.

## Figures and Tables

**Table 1:** Details of inclusion and exclusion criteria according to the study group.

Group	Identified	Excluded	Included
NTG	100 patients: NTG confirmed by a glaucoma specialist. Diurnal IOP lower than 21 mmHg before and during treatment.	Five patients excluded due to a lack of willingness.	95 patients
HTG	100 patients: HTG confirmed by a glaucoma specialist. Diurnal IOP higher than 21 mmHg before and during treatment.	40 patients excluded: 17 patients did not want to participate in the study; 8 patients changed their telephone number or were not reachable; 9 patients had an artefact in ICP measurement; 4 patients had trabeculectomy; 1 patient underwent cataract surgery; 1 patient died.	60 patients
HSs	65 subjects: age-matched healthy adults with no history of glaucoma or other diseases that could bias the results.	Three subjects had an artefact in ICP measurement.	62 subjects

NTG: normal-tension glaucoma patients; HTG: high-tension glaucoma patients; HSs: healthy subjects; IOP: intraocular pressure; ICP: intracranial pressure.

**Table 2:** Composition of the study groups.

Group	Age (Mean ± SD), Years	Gender, Female, %	Glaucoma Surgery	Glaucoma Medications, *N*	Systemic Medications, *N*
NTG	57.52 ± 10.88	79	No	β blockers, 23 Pg analogues, 54 CAIs, 14 α2 agonists, 2	Diuretics, 8 β blockers, 23 ACE inhibitor, 25 ARBs, 4 Others, 38
HTG	57.47 ± 10.90	54	No	β blockers, 13 Pg analogues, 17 CAIs, 10 α2 agonists, 4	Diuretics, 8 β blockers, 14 ACE inhibitor, 17 ARBs, 4 Others, 22
HS	57.39 ± 10.62	60	No	β blockers, 0 Pg analogues, 0 CAIs, 0 α2 agonists, 0	Diuretics, 2 β blockers, 10 ACE inhibitor, 6 ARBs, 1 Others, 14

NTG: normal-tension glaucoma patients; HTG: high-tension glaucoma patients; HSs: healthy subjects; *SD*: standard deviation; *N*: number of subjects; CAIs: carbonic anhydrase inhibitors; ACE: angiotensin converting enzyme; ARBs: angiotensin II receptor blockers.

**Table 3:** Results of the ICP values and tests of data normality.

Group	ICP (Mean ± SD) mmHg	95% CI of the Mean	Med	Min	Max	K–S Test Value	*df*	*p*-Value	Skewness (SE)	Kurtosis (SE)
NTG *N* = 95	9.42 ± 2.83	8.84–10.00	9.25	3.21	15.79	0.05	95	0.200	−0.02 (0.25)	−0.63 (0.50)
HTG *N* = 60	8.11 ± 2.68	7.42–8.80	8.08	3.37	15.17	0.06	60	0.200	0.49 (0.31)	−0.27 (0.61)
HSs *N* = 62	10.73 ± 2.16	10.18–11.28	10.62	7.26	15.17	0.09	62	0.200	−0.29 (0.30)	−0.89 (0.60)

NTG: normal-tension glaucoma patients; HTG: high-tension glaucoma patients; HSs: healthy subjects; *SD*: standard deviation; CI: confidence interval; Med: median; K–S test: Kolmogorov–Smirnov test; *df*: degrees of freedom; *SE*: standard error.
